# Retinal nerve fibre layer thickness measured with SD-OCT in a population-based study: the Handan Eye Study

**DOI:** 10.1136/bjophthalmol-2021-320618

**Published:** 2022-04-05

**Authors:** Jian Wu, Yifan Du, Caixia Lin, Yingting Zhu, Wei Chen, Qing Pan, Yehong Zhuo, Ningli Wang

**Affiliations:** 1 Beijing Institute of Ophthalmology, Beijing Tongren Eye Center, Beijing Tongren Hospital, Capital Medical University; Beijing Ophthalmology & Visual Sciences Key Laboratory, Beijing, China; 2 Department of Ophthalmology, Stanford University School of Medicine, Palo Alto, California, USA; 3 State Key Laboratory of Ophthalmology, Zhongshan Ophthalmic Center, Sun Yat-sen University, Guangzhou, China; 4 Department of Epidemiology and Biostatistics, West China School of Public Health and West China Fourth Hospital, Sichuan University, Chengdu, Sichuan, China

**Keywords:** retina, epidemiology, optic nerve

## Abstract

**Purpose:**

To examine the normative profile of retinal nerve fibre layer (RNFL) thickness and ocular parameters based on spectral-domain optical coherence tomography (SD-OCT) and its associations with related parameters among the Chinese population.

**Methods:**

This population-based cohort Handan Eye Study (HES) recruited participants aged≥30 years. All subjects underwent a standardised ophthalmic examination. Peripapillary RNFL thickness was obtained using SD-OCT. Mixed linear models were adopted to evaluate the correlation of RNFL thickness with ocular parameters as well as systemic factors. R V.3.6.1 software was used for statistical analysis.

**Results:**

3509 subjects (7024 eyes) with the average age of 55.54±10.37 were collected in this analysis. Overall mean RNFL thickness measured was 113.46±10.90 µm, and the thickest quadrant of parapapillary RNFL was the inferior quadrant, followed by the superior quadrant, the nasal quadrant and the temporal quadrant. In the multivariate linear regression model, thinner RNFL thickness was remarkable association with male (p<0.001), older age (p<0.001), increased body mass index (>30, p=0.018), absence of diabetes (p=0.009), history of cataract surgery (p=0.001), higher intraocular pressure (p=0.007), lower spherical equivalent (p<0.001) and increased axial length (p=0.048).

**Conclusions:**

In non-glaucoma individuals, this difference of RNFL thickness in Chinese population should be noted in making disease diagnoses. Meanwhile, multiple ocular and systemic factors are closely related to the thickness of RNFL. Our findings further emphasise the need to demonstrate ethnic differences in RNFL thickness and the specificity of associated ocular and systemic factors, as well as to develop better normative databases worldwide.

**Trial registration number:**

HES was registered in Chinese Clinical Trial Registry website, and the registry number was ChiCTR-EOC-17013214.

**Key messages**

**What is already known on this topic**

- The thickness of retinal nerve fibre layer (RNFL) is associated with varieties of eye diseases, which had been investigated in previous studies. However, lack of population-based studies in Asian population reported the RNFL thickness normative profile.

**What this study adds**

- This study explored the distribution of the RNFL thickness in the representative Chinese population.
- Meanwhile, the results conducted that RNFL thickness in our study was thicker than European population considering the ethnic difference.
- What’s more, the study demonstrated that the ocular and systemic factors were closely affecting RNFL thickness, like examination instruments, age, gender and axial length.

**How this study might affect research, practice or policy**

- Using the normative European database of RNFL thickness to measure the Chinese population may lead to misdiagnosis, especially glaucoma and neurodegenerative disease.
- The influence of various factors on RNFL thickness should be well considered during the eye disease diagnosis procedure.
- For achieving the goal of precision medicine, the results emphasise the critical need to establish a better normative database that included a multiethnic population worldwide.

## Introduction

Glaucoma is a leading cause of blindness, with a prevalence of 3.54% worldwide. Glaucoma is characterised by degeneration of retinal ganglion cells, which results in thinning of the retinal nerve fibre layers (RNFL) and irreversible visual field damaged.^1^ Several studies indicated that approximately half of the retinal nerve fibres may loss before occurrence of visual field defects in glaucoma patients.^2 3^ Therefore, the assessment of RNFL thickness is important in the early detection and diagnosis of glaucoma.

Spectral-domain optical coherence tomography (SD-OCT) is a noninvasive imaging technique that used to evaluate the RNFL and optic disc with high resolution cross-sectional^4^ retinal images.^5^ As SD-OCT technology grows gradually and the sufficient resolution images conduct the automated segmentation and measurement of individual retinal layer clearly, the evaluation of RNFL thickness has been extensively applied and involved in clinical practice for glaucoma diagnosis and follow-up. Meanwhile, as the retina is a laminated constituent of the central nervous system, it is the only part of the central nervous system which can be imaged optically with non-invasive methods, RNFL thickness measurements is also considered as a biomarker for neurodegenerative diseases, such as Parkinson’s^6^ and Alzheimer’s^7^ disease.

The normative database of RNFL thickness on SD-OCT was built in European, considering the parameters are different between each ethnic population,^8 9^ several studies around the world have been dedicated to eye diseases. Most of the research on OCT-based examinations are usually derived from relatively small sample populations,^10 11^ or assessed in hospital-based studies, and few of them have described RNFL thickness distribution in the healthy population-based setting, giving rise to potential selection bias.^12^

Since knowledge of RNFL thickness and optic nerve parameters are important for detection of glaucoma and other possible neurodegeneration diseases, the aim of this study was to investigate the distribution of peripapillary RNFL (pRNFL) thickness in the population-based Handan Eye Study (HES). In addition, by analysing the correlation of RNFL with age, gender and related ophthalmic parameters to further identify the potential risk factors of glaucomatous fundus damage.

## Material and methods

### Study population

The HES is a population-based cohort study that recruited subjects with an age of 30+ years residing in North China. Our study population was made up of 6830 individuals from the baseline visit in 2006–2007, 5394 participated the 6-year follow-up visit in 2012–2013 (response rate 85.3%). Without undergoing SD-OCT assessments, the baseline visits were not included in the study. The details about the study have been illustrated previously.^13^

The OCT data from 4037 participants (7950 eyes) were available except the participants without OCT measurements. Of them, 425 eyes were further excluded due to poor OCT images quality with signal strength less than 45. Meanwhile, 177 eyes with glaucoma, retinal disease or history of ocular surgery were also excluded. Glaucoma was diagnosed by three aspects: the eye fundus, the visual field results, and intraocular pressure (IOP), and finally defined according to the standardised International Society of Geographical and Epidemiological Ophthalmology criteria.^14^ Therefore, the research enrolment a total of 4766 eyes of 2638 participants.

### Ocular and systemic examination

All participants underwent a comprehensive clinical examination by ophthalmologists receiving standardised training. A detailed interviewer questionnaire was conducted to collect demographic variables, educational background, a medical history consisting of smoking and known major systemic diseases information. Body mass index (BMI) was calculated as the ratio of body weight (in kilograms) divided by body height (in metres) squared. Hypertension was defined as systolic blood pressure ≥140 mm Hg or diastolic blood pressure ≥90 mm Hg, or use of antihypertensive medications.^15^ Diabetes was defined as fasting plasma glucose ≥7.0 mmol/L, self-reported diagnosis of diabetes or the use of antidiabetic medications.^16^ Coronary heart disease was defined as self-reported coronary heart disease, stroke or related peripheral artery disease. Blood samples were collected for measurements of high-density lipoprotein, low-density lipoprotein and triglyceride concentration.

Slit-lamp biomicroscopy was performed by experienced ophthalmologists and IOP was measured using the Kowa applanation tonometer (HA-2, Kowa Company, Tokyo, Japan). Best-corrected visual acuity (BCVA) was tested monocularly (right eye followed by left eye) then using a log MAR chart at a distance of 4 m binocularly. A 10 MHz A/B-mode ultrasound device was used (Cine Scan, Quantel Medical, Clermont-Ferrand, France) to measure axial length (AL). Autorefractor (KR8800, Topcon, Tokyo, Japan) was applied to measure the refraction and corned curvature on the occasion of no pupil dilation.

### OCT Imaging

pRNFL parameters in both eyes were measured by SD-OCT (RTVue 100-2, Optovue, Fremont, California, USA; V.4.0). The device uses a scanning laser diode to emit infrared light-source with wavelength of 840 nm and acquires 26 000 A-scans per second scan. The position of the aiming circle was adjusted by the experienced examiner to match the optic nerve head (ONH), and the 12 radial scans (scans ranging from 1.3 to 4.9 mm) was made covering a measurement area of 3.4 mm diameter ring that included the ONH and surroundings in all directions. Various ONH and RNFL parameters (disc, cup and rim area, cup and ONH volume, cup/disc ratio, average and vertical cup-to-disc ratios, average and per-quadrant pRNFL thickness) were measured by algorithms native of RTVue OCT automatically. The outputs parameters included (1) average RNFL thickness; (2) temporal, superior, nasal, and inferior average RNFL thickness; and (3) 16 sections of the measuring circle around the ONH (each section was 22.58). OCT scans images quality is described by the signal strength index (SSI), which is based on the intensity of the reflected light. The SSI ranged from near 0 (no signal) to approximately 90 (very strong signal), algorithm segmentation failure and obvious decentration misalignment were excluded further from the analysis with SSI less than 45 (as recommended by the manufacturer).

### Statistical methods

Data analysis was carried out by using the statistical software R (V.3.6.1; R Core Team, 2019). The quantitative data were represented in mean and SD (or median, IQR) and qualitative data was described with counts and percentage. χ^2^ test, t-test, and one-way analysis of variance (ANOVA), Turkey tests as post hoc tests after ANOVA were applied to detect the difference between groups. Bonferroni correction to control for the potential false discovery in multiple comparisons. Variance inflation factor (VIF) for each covariate in the multivariate model was conducted, a linear mixed model was used to estimate the relationship between individual features and RNFL. The significant level was set as p<0.05.

## Results

### Comparisons of characteristics between the excluded and included individuals

#### Study population

The demographic characteristics in the study population are described in table 1. In total, 7024 eyes of 3509 participants were included in the analysis (1912 men and 1607 women). The age ranged from 35 to 86 with an average of 55.54±10.37 years. Compared with excluded group, the included subjects tended to be significantly younger (p<0.001), higher body height and weight (p<0.001), higher systolic (p<0.001), absence of diabetes and lower glycosylated hemoglobin (HbA1c) (p<0.001), less coronary heart disease (p<0.001), higher cataract extraction (p<0.001) and lower BCVA (p<0.001) (table 1).

**Table 1:** Comparisons of demographic and biochemical characteristics between the included and excluded individuals

Variables	Excluded group (N=1885)	Included group (N=3509)	T/χ^2^	P value
Age (years)	59.56±11.79	55.54±10.37	12.446	**<0.001**
Sex (men, %)	789 (41.9)	1617 (46.1)	8.688	**0.003**
High educational level				
Lower than high school	1843 (97.8)	3394 (96.7)	4.777	**0.029**
High school or above	42 (2.2)	115 (3.3)		
Smoking habits				
Yes	409 (23.6)	916 (26.6)	5.250	**0.022**
No	1326 (76.4)	2533 (73.4)		
Body mass index (kg/m^2^)	25.78±4.06	25.92±3.88	−1.199	0.231
Body height	158.49±8.46	159.76±8.05	−5.247	**<0.001**
Body weight	64.93±11.51	66.19±11.22	−3.810	**<0.001**
Blood pressure diastolic (mm Hg)	83.66±13.51	84.09±12.74	−1.120	0.263
Blood pressure systolic (mm Hg)	145.60±23.63	141.63±21.52	6.041	**<0.001**
Hypertension				
Yes	593 (35.3)	1015 (30.5)	11.855	**0.001**
No	1085 (64.7)	2317 (69.5)		
Diabetes				
Yes	123 (7.4)	128 (3.9)	27.739	**<0.001**
No	1538 (92.6)	3167 (96.1)		
HbA1c (%)	5.87±1.05	5.71±0.75	5.834	**<0.001**
High-density lipoprotein cholesterol (mmol/L)	1.20±0.27	1.22±0.28	−2.426	**0.015**
Low-density lipoproteins (mg/dL)	2.71±0.76	2.65±0.74	2.629	**0.009**
Triglycerides (mg/dL)	1.47±1.12	1.40±1.14	2.049	**0.041**
Coronary heart disease				
Yes	183 (11.0)	249 (7.6)	37.880	**<0.001**
No	1486 (89.0)	3027 (92.4)		
Cataract extraction (OS)				
No	1801 (97.6)	3481 (99.4)	30.600	**<0.001**
Yes	44 (2.4)	22 (0.6)		
Cataract extraction (OD)				
No	1797 (97.6)	3480 (99.3)	27.935	**<0.001**
Yes	44 (2.4)	24 (0.7)		
Intraocular pressure OS (mm Hg)	12.32±2.70	12.31±2.29	0.091	0.927
Intraocular pressure OD (mm Hg)	11.76±2.91	11.80±2.32	−0.457	0.648
Spherical equivalent OS	0.06±2.09	0.11±1.76	−1.017	0.309
Spherical equivalent OD	0.06±1.97	0.07±1.84	−0.199	0.842
Axial length OS (mm)	22.78±0.95	22.82±0.91	−1.538	0.124
Axial length OD (mm)	22.85±0.98	22.87±1.08	−0.793	0.428
BCVA OS (logMAR)	0.60±0.26	0.56±0.26	4.437	**<0.001**
BCVA OD (logMAR)	0.60±0.26	0.57±0.26	3.570	**<0.001**

Statistics presented: mean±SD; n (%).

Values with statistical significance are shown in boldface.

BCVA, best-corrected visual acuity; OD, Oculus Dexter; OS, Oculus Sinister.

### Distribution of RNFL thickness

**Figure 1:**
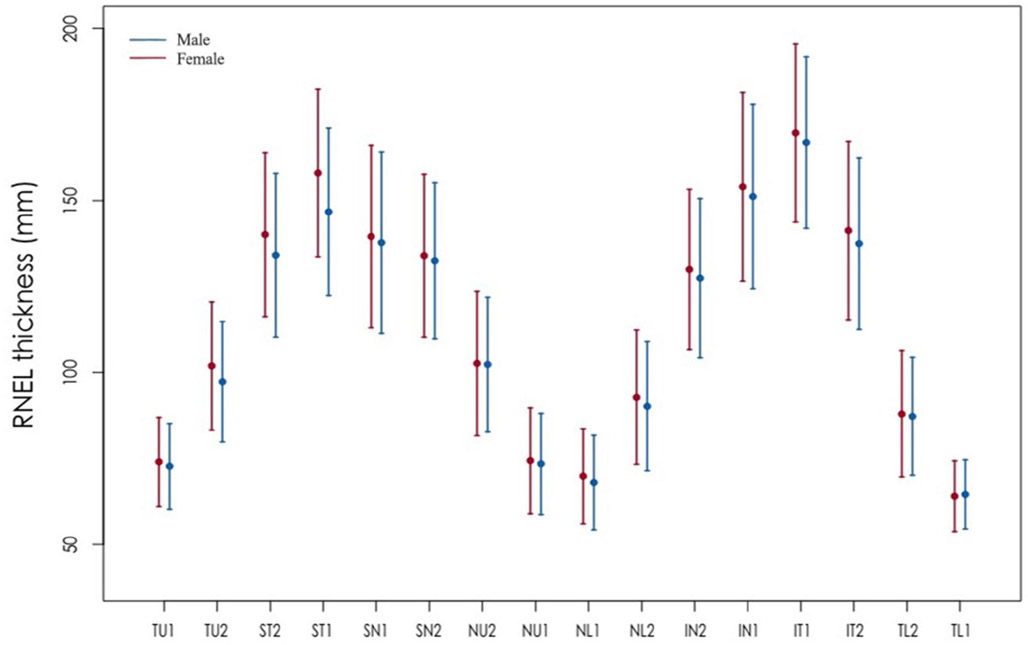
Distribution of RNFL thickness by gender. This figure shows the distribution of RNFL in 16 segments near the optic disc of different genders. It can be seen that the distribution of RNFL thickness was the same as the overall. The RNFL of 16 segments was all thicker in women than in men. RNFL, retinal nerve fibre layer.

The average RNFL thickness in this study was 113.46±10.90 µm. And the thickest quadrant of pRNFL was the inferior quadrant, followed by the superior quadrant, the nasal quadrant and the temporal quadrant. The mean RNFL thickness was 2.11 µm thicker in female than in male (p<0.001). These sex-specific differences were statistically significant also in all temporal, nasal, superior and inferior (p<0.001) quadrants. All segments were thicker in female than in male, as shown in figure 1. For the optic disc parameters, the mean (SD) disc, cup and rim area were 2.14 (0.44) mm, 0.67 (0.39) mm and 1.46 (0.44) mm, respectively. Compared with male, female had significant smaller average cup-to-disc ratio (p<0.001), cup volume (p<0.001), AL (p<0.001) and larger rim area (p<0.001) (online supplemental tables 2 and 4).

10.1136/bjophthalmol-2021-320618.supp1 Supplementary data

### The relationship between RNFL and age

**Figure 2:**
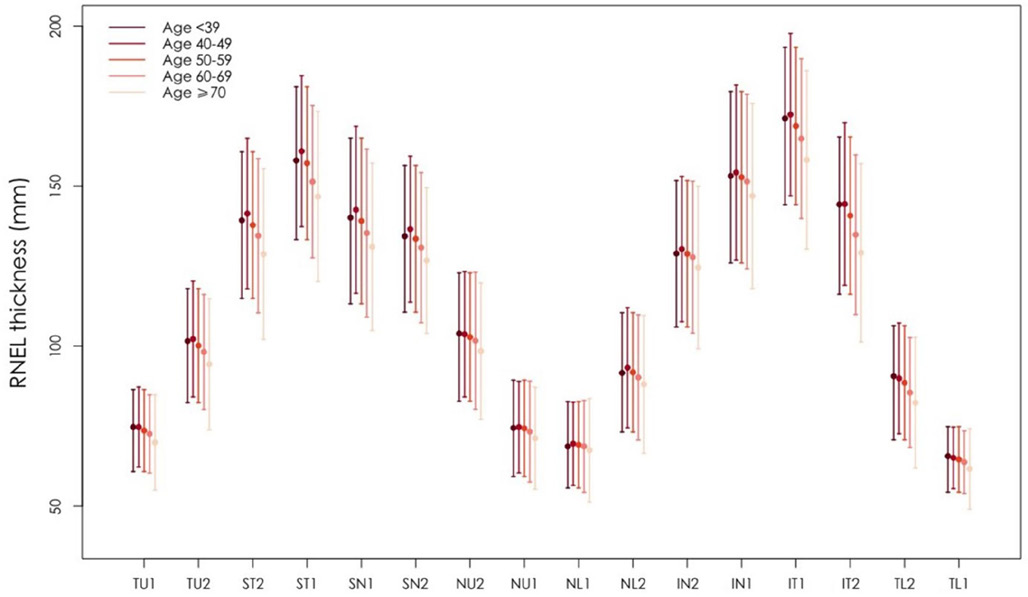
Distribution of RNFL thickness at different ages. This figure shows the distribution of RNFL in 16 segments near the optic disc at different genders. It can be seen that the thickness quadrant of RNFL is the inferior quadrant, followed by the superior, nasal and temporal quadrant. The thickness of peripheral RNFL decreases with age but this trend is gradually reflected after 50 years old. RNFL, retinal nerve fibre layer.

**Figure 3:**
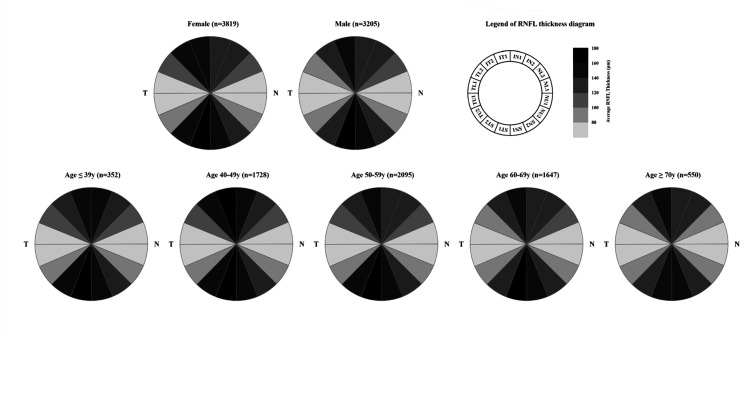
Fan-shaped distribution of RNFL thickness by gender and different ages. The fan-shaped distribution can show that the RNFL thickness near the optic disc is the thinnest in TL1, TU1, NI1 and NU1 and the thickest in ST1 and IT1. The fan-shaped distribution of RNFL thickness showed a thinner trend in men and older people. RNFL, retinal nerve fibre layer.

The normative distribution of RNFL thickness by age groups is described in table 2. Participants were divided into five age groups: less than 39, 40–49, 50–59, 60–69, over 70 years. For all age-specific groups, all inferior quadrant RNFL mean thickness was thicker than superior quadrant, followed by nasal and temporal quadrant mean RNFL. The four quadrants thickness sectors are shown in table 2. Mean RNFL thickness was highest in the less than 39-year-old age group (115.22 um) vs 107.70 um in the over 70-year-old age group. A general trend toward thinner RNFL was noted with older age in 16 sectors is show in figure 2 and online supplemental table 3. Of ONH parameters, AL decreased and disc area increased significantly with age (table 2), the post hoc tests after ANOVA among the age groups were shown in online supplemental tables 7 and 8. Mixed linear regression models were used to compare the sectionalised RNFL thickness between different age and gender groups. And the fan-shaped distribution of RNFL regions of different ages and genders was shown in figure 3. With the main and interaction effect from age and gender were both considered, the results demonstrated that except for RNFL, the coefficients of interaction effect were non-significantly (online supplemental tables 1 and 3).

**Table 2:** Comparisons of sectionalised RNFL thickness between different age groups

Segment	Age					
	<39 (N=352)	40–49 (N=1728)	50–59 (N=2095)	60–69 (N=1647)	≥70 (N=550）	P value
RNFL thickness (μm)						
Average	115.22±10.88	115.90±10.25	113.95±10.34	111.47±10.75	107.80±12.54	**<0.001**
Superior	143.28±19.00	145.29±17.89	141.81±17.76	137.96±18.39	133.22±19.47	**<0.001**
Nasal	85.05±14.78	85.14±14.63	84.53±15.01	83.42±16.05	81.23±16.22	**<0.001**
Inferior	149.61±17.31	150.14±17.27	147.73±16.89	144.67±17.50	139.40±19.56	**<0.001**
Temporal	83.06±12.54	83.03±12.27	81.76±12.32	79.84±12.00	76.98±14.60	**<0.001**
ONH parameters						
Rim area (mm^2^)	1.69±0.38	1.68±0.47	1.69±0.47	1.67±0.49	1.71±0.55	0.615
Disc area (mm^2^)	2.37±0.43	2.39±0.48	2.43±0.51	2.44±0.48	2.49±0.53	**<0.001**
Average CDR (mm^2^)	0.33±0.13	0.33±0.13	0.34±0.13	0.34±0.13	0.33±0.14	0.549
Cup volume (mm^3^)	0.13±0.15	0.15±0.19	0.15±0.18	0.14±0.17	0.13±0.17	0.228
Axial length (mm)	22.95±1.19	22.98±0.94	22.83±0.93	22.75±0.86	22.69±0.80	**<0.001**

Statistics presented: mean±SD; n (%).

Values with statistical significance are shown in boldface.

CDR, cup-to-disc ratio; ONH, optic nerve head; RNFL, retinal nerve fibre layer.

### Linear regression analysis of influencing factors of RNFL

Univariate and multivariate linear regression models were used to estimate the independent associations between ocular (history of cataract surgery, IOP, spherical equivalent (SE), AL, BCVA) and systemic (age, gender, educational level, history of smoking, BMI, hypertension and diabetes status, and LDL cholesterol) parameters with average RNFL thickness (table 3). In univariate linear regression analysis, thinner RNFL thickness was significantly associated with older age (p<0.001), male sex (p<0.001), absence of smoking (p<0.001), increased BMI value (BMI <21, p=0.017), increased BMI value (BMI >30, p=0.033), absence of diabetes (p=0.002), coronary heart disease (p<0.001), history of cataract surgery (p=0.001), higher IOP (p=0.043), lower SE (p<0.001), longer AL (p<0.001) and BCVA (p<0.001) (table 3).

**Table 3:** The univariate and multivariate analysis of relationship between demographic and biochemical characteristics and average RNFL thickness

Variables	Univariate analysis			Multivariate		
	B	SE	P value	B	SE	P value
Age (years)	−0.233	0.016	**<0.001**	−0.260	0.024	**<0.001**
Gender (female vs male)	2.043	0.346	**<0.001**	1.772	0.495	**<0.001**
High educational level	−0.576	0.973	0.554			
Smoking status	1.369	0.394	**<0.001**	−0.319	0.552	0.563
Body mass index (kg/m^2^)*						
<21	−1.531	0.643	**0.017**	−0.822	0.690	0.234
27–30	0.745	0.426	**0.080**	0.668	0.461	0.147
≥30	1.144	0.537	**0.033**	1.403	0.593	**0.018**
Diastolic >140 (mm Hg)	2.287	7.222	0.751			
Systolic >90 (mm Hg)	7.903	5.897	0.180			
Diabetes	2.888	0.929	**0.002**	2.576	0.992	**0.009**
HbA1c (%)	−0.412	0.516	0.425			
HDL (normal vs abnormal)	0.028	0.383	0.942			
LDL (normal vs abnormal)	0.414	0.368	0.260			
TG (normal vs abnormal)	0.165	0.398	0.678			
Coronary heart disease	2.248	0.677	**<0.001**	0.562	0.706	0.426
Cataract extraction	−4.591	1.419	**0.001**	−5.845	1.633	**<0.001**
Intraocular pressure (mm Hg)	−0.110	0.054	**0.043**	−0.157	0.059	**0.007**
Spherical equivalent	0.734	0.076	**<0.001**	0.862	0.108	**<0.001**
Axial length	−0.513	0.112	**<0.001**	−0.240	0.121	**0.048**
BCVA (logMAR)	−4.270	0.593	**<0.001**	−0.703	0.724	0.331

Values with statistical significance are shown in boldface.

*21-27 (kg/m^2^) was set as the reference category.

BCVA, best-corrected visual acuity; HbA1c, glycosylated hemoglobin; HDL, high density lipoprotein; LDL, Low Density Lipoprotein; RNFL, retinal nerve fibre layer; TG, triglyceride.

In the multivariate analysis model, RNFL thickness was taken as the dependent variable, the significant (p<0.05) variables in univariate models were further included in the multivariate mixed linear model (table 3). We then dropped the variables that were no longer significantly associated with average RNFL thickness. In the multivariate model, thinner RNFL thickness was significantly associated with older age (p<0.001), male sex (p<0.001), increased BMI value (BMI >30, p=0.018), absence of diabetes (p=0.009), history of cataract surgery (p=0.001), higher IOP (p=0.007), lower SE (p<0.001) and longer AL (p=0.048). In addition, we supplemented the VIF for each covariate in the multivariate model, the VIFs were all below 10 (online supplemental table 5).

## Discussion

We evaluated the RNFL thickness around the optic disc and the parametric distribution of pRNFL measured by SD-OCT among the non-glaucomatous Asian population. This is the first population-based cohort study in China and showing the discrepancy in the standard distribution of RNFL thickness in middle-aged and older populations. Quadrant thicknesses decreasing order: inferior, superior, nasal and temporal, respectively. We demonstrated that the RNFL was significantly thicker in the normal Asian population than in the European standard database using SD-OCT. We demonstrated that the RNFL thickness tends to be influenced by races. Therefore, errors may occur in measuring Asian individuals when the RNFL thickness standard database composed of other races (such as Caucasians) in OCT measurement. Moreover, various factors may also affect the RNFL thickness evaluation. RNFL thickness thinned with age, while women showed a tendency to be thicker in RNFL measurements. And other factors such as IOP, AL, cataract surgery, diabetes and others were also shown to be associated with RNFL thickness.

This study is a population-based cohort study (all data based on HES), which was statistically analysed to yield a mean RNFL thickness for this in Chinese population, and the average RNFL thickness of Chinese population is 113.46±10.90 µm. The distribution of RNFL thickness was consistent with the distribution of RNFL in the majority of studies. As described by Zhao *et al* 17 measuring RNFL in subjects without optic nerve or retinal disease, the average RNFL was significantly higher in the inferior temporal quadrant than in the superior temporal quadrant, followed by the inferior nasal quadrant, the superior nasal quadrant, the temporal region and finally the nasal quadrant, respectively. This sequence of nasal quadrants bore a definite resemblance to the Singapore-Malay study^18^ and the Singapore Chinese Eye Study.^19 20^ However, the mean thickness of RNFL in our study population was thicker compared with the most of other studies. In the Gutenberg Health Study,^21^ the overall mean thickness of RNFL was 96.0 µm, and the Singapore Chinese Eye Study^19 20^ showed an overall mean RNFL thickness of 96.2 µm. Another population-based cohort study from France found a mean RNFL thickness of 91.4 µm,^22^ all these figures were obviously lower than our findings. In contrast, the overall average pRNFL thickness in the Beijing Eye Study was 103.2 µm (SD-OCT), which shown similar results as our study, although it is also lower than our results. The differences in RNFL measurements can be partially explained by the different instruments applied and various age groups of the population covered. However, even for the same examination equipment, the RNFL thickness in our study population compared with other European or African populations is still thicker.^23^ The RNFL thickness of other studies was shown in table 4,^8 9 17 19 21 22 24–54^ it can be roughly seen that the RNFL thickness measured by Cirrus OCT is generally lower than that measured by other OCT equipment according to this table, which may come from the differences in the built-in RNFL thickness standard database of OCT machines by various companies. The reason for the differences is that these companies shall select diverse individuals with different proportions when setting the standard database of the RNFL thickness. For instance, Heidelberg company selects all Caucasians, Topcon selects all Asians, and other companies select a wide range of people (online supplemental table 6). Thus, when the difference of RNFL thickness on account of various ethnic groups is judged, the database of the instrument itself must show the certain impact, suggesting that the instrument differences shall be taken into consideration in judging RNFL thickness in ethnic groups. Therefore, the international standard RNFL database is not suitable for the application in the Chinese population, which may bring about a lot of missed diagnosis (taking the glaucoma as the normal one), or misdiagnosis (taking normal one as glaucoma) potentially, suggesting the need to establish additional appropriate RNFL thickness measures in the Chinese population.

**Table 4:** Information of RNFL thickness from previous studies

Study	Year	OCT	Country	N	Age	RNFL thickness (μm)				
						Average	Temporal	Superior	Nasal	Inferior
Girkin *et al* 24	2010	Stratus OCT	Africa	315	45.1±13.3	103.7±10.7	66.5±11.1	128.8±17.2	84.3±17.2	135.1±16.3
Girkin *et al* 24	2010	Stratus OCT	Europe	290	47.7±15.9	100.6±10.9	71.5±12.6	120.9±17.5	80.8±16.3	129.2±17.4
Celebi and Mirza^25^	2013	Cirrus SD-OCT	Turkey	121	38.9±11.2	97.0±7.4	64.7±6.4	119.2±11.2	74.8±8.2	129.3±11.7
Alasil *et al* 26	2013	Spectralis OCT	Mixed	190	53.7±16.3	97.3±9.6	70.6±10.8	117.2±16.3	75.0±14.0	126.0±15.8
Park *et al* 27	2005	Stratus OCT	Korea	121	43.2±13.9	112.7±15.0	85.2±17.9	137.5±20.0	89.5±22.2	138.1±20.8
Budenz *et al* 28	2007	Stratus OCT	America	328	47.4±15.8	100.1±11.6				
Schuster *et al* 29	2016	Topcon 3D-OCT	Caucasian	306	38.8±10.9		87.0±11.0	124.0±13.0	93.0±15.4	138.0±13.6
Cheung *et al* 19	2011	Cirrus HD-OCT	Chinese	542	53.0±6.4	97.6±9.1	71.6±11.2	123.0±15.9	69.2±10.8	126.8±16.2
Sung *et al* 30	2009	Stratus OCT	America	226	47.5±15.9	100.8±10.5				
Leung *et al* 31	2004	Stratus OCT	China	107	53.0±11.8	103.2±10.0				
Kanamori *et al* 32	2003	Humphrey OCT	Japan	144	46.3±18.1	123.0±11.6	101.0±18.5	148.0±18.4	96.0±19.2	146.0±19.3
Fujiwara *et al* 9	2019	Topcon 3D-OCT	Japan	749	58.0±10.0	102.3±0.9				
Zangalli *et al* 33	2018	Spectralis OCT	Brazil	220	44.0±13.9	103.0±10.4	67.7±10.6		86.0±13.8	
Perez *et al* 34	2018	Cirrus OCT	Vietnam	151	60.8±11.1	97.9±9.2	69.7±10.1	119.7±15.1	73.4±13.8	128.6±15.4
Manassakorn *et al* 35	2008	Stratus OCT	Thailand	250	44.7±12.2	109.3±0.7	75.1±0.7	136.0±1.0	83.6±1.0	142.4±1.1
Appukuttan *et al* 36	2014	Spectralis OCT	India	150	20–75	101.4±8.6	72.0±7.7	125.3±13.7	79.7±12.1	128.3±14.7
Al-Sa'ad *et al* 37	2018	RTVue OCT	Jordan	148	60.0±12.0	99.0±11.0	82.0±20.0	114.0±20.0	75.0±16.0	125.0±20.0
Varma *et al* 38	2003	OCT 2000	Latino	312	51.9±9.8	132.7±14.4	102.5±19.0	157.7±17.8	109.3±19.1	159.8±18.9
Kang *et al* 39	2016	iVue-100 OCT	China	1811	7.1±0.4	102.0±0.2	80.2±0.2	125.1±0.3	75.9±0.3	126.8±0.3
Méndez *et al* 40	2017	Spectralis OCT	France	427	81.6±4.2	86.8±13.7	67.2±13.4		66.2±16.4	
Kanno *et al* 41	2010	EG-SCANNER	Japan	460	44.0±14.5	111.8±10.0				
Gupta *et al* 42	2015	Cirrus HD-OCT	Singapore	843	66.7±10.4	92.9±11.4	67.4±11.4	116.1±18.4	69.7±11.3	118.6±19.8
Rougier *et al* 22	2015	Spectralis OCT	French	210	81.0±3.6	91.4±12.6	70.1±14.7		69.1±16.3	
Ho *et al* 43	2019	Cirrus HD-OCT	Chinese	1371	57.4±7.0	95.7±9.6	71.4±12.2	119.9±16.7	68.1±10.9	123.4±16.7
Ho *et al* 43	2019	Cirrus HD-OCT	Malay	1303	60.6±8.6	94.9±10.6	67.8±11.3	118.6±17.3	71.0±10.9	122.4±18.3
Ho *et al* 43	2019	Cirrus HD-OCT	Indian	1801	60.7±7.8	87.3±10.6	59.2±10.9	108.8±16.5	69.1±11.1	112.3±17.3
Zhu *et al* 44	2013	iVue-100 OCT	China	1955	12.3±0.6	103.1±9.0	83.0±10.6	126.2±15.2	73.8±13.9	129.3±14.9
Wang *et al* 45	2018	Spectralis OCT	China	1440	11.9±3.5	101.3±9.2	85.2±14.3		61.7±20.4	
Nousome *et al* 46	2021	Cirrus HD-OCT	Mixed	6133	60.1±7.4	95.1±10.1	65.6±12.2	118.6±16.5	71.2±11.1	125.1±17.3
Malik *et al* 47	2012	Stratus OCT	India	150	42.6±13.6	101.1±10.1	65.7±12.1	125.8±16.5	83.6±17.4	127.5±15.6
Bendschneider *et al* 8	2010	Spectralis OCT	America	170	20–78	97.2±9.7	68.8±11.1	118.0±14.5	76.4±15.0	123.7±16.4
Wang *et al* 48	2013	iTVue OCT	China	1654	66.2±9.9	103.2±12.6	79.8±12.2	126.1±19.1	75.1±12.6	131.4±20.6
Hashemi *et al* 49	2017	Cirrus HD-OCT	Iran	3084	54.3±5.6	92.5±0.3	65.5±0.4	111.2±0.5	74.8±0.8	118.9±0.6
Thapa *et al* 50	2014	Spectralis OCT	Nepal	156	38.9±17.0	102.6±9.6	70.7±15.5	129.5±15.1	76.6±12.0	134.5±17.2
Ismail *et al* 51	2019	Spectralis OCT	South Africa	132	41.3±12.5	108.7±10.7	74.8±10.3		77.7±14.6	
Zhao *et al* 17	2014	Spectralis OCT	China	2548	63.5±9.1	102.0±11.0	76.0±13.0		73.0±15.0	
Mashige and Oduntan^52^	2016	iVue-100 OCT	South Africa	600	10–66	110.0±7.4	73.6±15.7	132.0±10.5	87.2±13.2	135.1±9.7
Budenz *et al* 53	2005	Stratus OCT	America	109	42.8±14.6	104.8±10.7	75.1±17.2	130.9±18.2	79.8±17.2	133.4±18.7
Hoffmann *et al* 21	2018	Spectralis OCT	Germany	1974	40–80	96.0±10.3	68.8±12.9		72.2±15.1	
Feuer *et al* 54	2011	Stratus OCT	America	425	46.0±15.0	104.7±10.8				

RNFL, retinal nerve fibre layer; SD-OCT, spectral-domain optical coherence tomography.

Age is one of the important factors affecting the distribution of RNFL thickness, which has been confirmed in several studies. In the study conducted by Hashemi *et al*, the overall average RNFL thickness as well as the mean thickness of all quadrants went down dramatically with age,^49^ while the decrease of RNFL thickness^8 25^ was in connection with the ageing in other studies also. In addition to OCT studies, some studies have shown age-related RNFL thinning by histological analysis.^26^ The RNFL thickness decreases by 2–4 µm per 10 years of ageing has been observed in individuals over 50 years, whereas it nearly hasn’t been found under 50 years old or even younger people in accordance with these results. The rationale for the effect of age on the RNFL may be due to decreasing blood supply to the fundus and senescence apoptosis of the optic nerve with advancing age.^21^ The additional systemic factors effecting the measurement of RNFL thickness shall not be ruled out as the age grows. Nevertheless, there definitely exists the correlation of a decrease in RNFL thickness with an increase in age. Therefore, RNFL thickness for different age groups may need to be considered separately, and synthesising data from multiple studies and deriving a pattern of decay in RNFL thickness with age in order to advance the use of measuring RNFL thickness in clinical practice (figure 3).

Our study also found that women tended to be thicker than men in both overall average RNFL and RNFL thickness in all orientations. Rougier *et al* found that women tended to have a thicker RNFL than men overall and across all temporal ranges, although this was only significant in the inferior temporal segment.^22^ Even higher RNFL values in females and even female children have been reported in some population-based studies and clinical-based studies.^55^ However, some studies have failed to find an association between RNFL and gender in adult populations, as recently Girkin *et al* 23 found that pRNFL or macular parameters obtained using SD-OCT (RTVue) did not differ between females and males. Although this correlation needs to be explored in further studies, however, the gender should be taken into consideration when the RNFL is measured and analysed (figure 3).

As for the correlation analysis of systemic factors with RNFL, the lower BMI (BMI ≥30) and no diabetes were correlated linearly with RNFL thinning in both univariate and multivariate linear analyses. In terms of the BMI, the higher BMI is related to the increase of intracranial pressure, and the increase of intracranial pressure can lead to changes such as optic disc oedema, making the measured RNFL thicker,^56^ then showing the lower BMI and the decline of RNFL are definitely related. Most of the studies reported thinner RNFL in patients with diabetes,^57^ which may be on account of diabetes-induced microangiopathy and ischaemia, and the correlation between reduced RNFL visibility and elevated blood glucose concentrations was associated with loss of retinal nerve fibres in patients with diabetic retinopathy.^58^ However, there is no evidence that diabetes is related to the thinning of RNFL in our study, which may be due to the proliferation and retinal oedema caused by diabetes. Our study did not find a linear relationship between elevated blood pressure as well as coronary artery disease and RNFL, although previous studies have also reported conflicting results regarding the effect of hypertension on RNFL.^59^ Nevertheless, the hypertensive patients facing with fluctuations in blood pressure or medication control may give rise to the negative results and hypertensive fundus lesions may affect RNFL thickness in our study according to our speculation. This study did not find an association between smoking and RNFL, Mauschitz *et al* still suggested such an association, although its relationship of smoking with increased or decreased RNFL is unclear.^60^ Other central nervous system conditions similar to stroke, apoplexy, and dementia have also been shown to be associated with RNFL thickness. Quite a few systemic conditions can affect RNFL thickness measurements, which is a factor that cannot be ignored when building a standard RNFL database. Broadly speaking, however, it appears that the vast majority of these systemic factors affect the status of vascular microcirculation throughout the body.

Generally, elevated IOP, decreased SE and AL were also connected with the declining of RNFL in both univariate and multivariate linear regression analyses after the cataract surgery. As for the IOP, a host of studies suggest that the decreasing of RNFL bears the association with higher IOP, which is one of the major potential factors for glaucoma after all.^10 31^ However, the study by Mauschitz *et al* also confirmed that high IOP was associated with a reduction in RNFL thickness even exclusion^60^ of the patients with known glaucoma. The association between a longer AL and a thinner RNFL is another finding of this study, which has been confirmed by previous studies,^61^whereas the relationship between AL and RNFL thickness was not the same across quadrants. Yoo *et al* showed that increased AL was associated with decreased RNFL thickness in the upper, lower and nasal quadrants and increased RNFL thickness in the temporal quadrant.^62^ Kang *et al* studied Chinese individuals living in Korea using SD-OCT and reported similar results except for the temporal quadrant observation.^63^ In a European study, Savini *et al* demonstrated a remarkable association that a larger AL was significantly associated with lower RNFL thickness in all quadrants. Whereas Hirasawa *et al* used a simple regression model to show that thickness was directly correlated with AL in all quadrants, while this kind of relation was only present in the temporal quadrant in their multiple regression model.^12^ Although the vast majority of studies have found a relationship between AL and RNFL, this relationship is nevertheless influenced by many factors. As for the SE, the decline in both SE and RNFL may always occur among the elder for the majority of them having the vision loss. Moreover, the ocular disease history is also a factor that affects RNFL and these factors have to be taken into account in the measurement of RNFL.

This study also found a relationship between cataract surgery and thinner RNFL thickness. We speculated that this should be due to the fact that the average age of people with cataract surgery (with average age 56 years) is much older than that of people without cataract surgery (with average age 70y). However, in addition to age, some studies have also proposed the relationship between cataract surgery and RNFL thickness. The macular and RNFL thickness face with a rise after cataract extraction partly on account of the quality of scanning image developing proposed by Pašová and Skorkovská,^64^ while the RNFL and macular measurement conduncted by SD-OCT shall be influential by the presence of cataract and a better OCT instrument may reduce this effect as much as possible suggested by Bambo *et al*.^65^ Therefore, the cataract surgery makes changes on the penetration of refractive medium, then further impacting the measurement of RNFL thickness.

Our study has several advantages. First, this large population-based study involved 3509 participants all coming from the general population instead of that attending the hospital. Second, the ocular examination excluded the participates with ocular disease, which made the RNFL measurements more accurate, and could be representative of the normal database. Third, the SD-OCT evaluation was performed by experienced technicians on the same machine following a standardised protocol, the bias of measurement has been avoided. And applied SD-OCT provided the best image quality and reproducibility currently available. Finally, we simultaneously evaluated many possible risk factors associated with RNFL thickness.

However, there are still some limitations of our study: although Chinese people were selected to explore the thickness of RNFL in this study, the thickness of RNFL also depends on other factors. As the rural population was selected in this study, the prevalence of myopia was relatively low for the participation of them in the study, and the AL (22.8 mm) was shorter than that in other studies involving Chinese population (Ho *et al* 43), further affecting the evaluation of the results. we cannot exclude the possibility that subjects with refractive media opacities could influence the results. However, routine clinical examinations would also face such a situation, which is unavoidable with SD-OCT examinations. Second, the assessment of RNFL visibility and local RNFL defects is subjective and therefore it depends on the technicians. In our study, however, all inspectors had been well trained, but it is inevitable that inspectors have varying experience and examination skills. Third, individuals excluded due to poor OCT scans were older and they were more likely to have hypertension, diabetes and hyperlipidaemia. Therefore, these biases could not be completely excluded in our final sample.

In conclusion, we measured the thickness and distribution of RNFL in the Chinese population, the results in our study demonstrate that the RNFL in the Chinese population is thicker than we widely use in normal SD-OCT database. Using the European RNFL database to evaluate the Chinese population may miss some glaucoma patients. Besides, it shall not be ignored in RNFL evaluation that various factors may have some influential on RNFL thickness and these related parameters also widely applied in the diagnosis of glaucoma and other neurological disorders. Hence, our findings further emphasise the need to demonstrate ethnic differences in RNFL thickness and the specificity of associated ocular and systemic factors, as well as to develop better normative databases worldwide.

10.1136/bjophthalmol-2021-320618.supp2 Supplementary data

## Data Availability

Data are available on reasonable request. Data may be obtained from a third party and are not publicly available.
