# Supplementary material for: Retinal nerve fibre layer thickness measured with SD-OCT in a population-based study: the Handan Eye Study
Source: Br J Ophthalmol. 2022 Apr 5;107(8):1156–64. doi: 10.1136/bjophthalmol-2021-320618 (PMC10359552; doi:10.1136/bjophthalmol-2021-320618)
Supplement: Supplementary data [file bjophthalmol-2021-320618supp001.pdf]

Supplemental table 1. Comparisons of RNFL thickness between age and gender subgroups

Segment	Age		Gender		Age*Gender	
	**B**	***P*-value**	**B**	***P*-value**	**B**	***P*-value**
**RNFL thickness (μm)**						
Average	-0.188	<0.001	3.597	0.047	-0.031	0.336
Superior	-0.245	0.003	5.742	0.047	-0.067	0.195
Nasal	-0.024	0.715	4.755	0.043	-0.061	0.140
Inferior	-0.278	<0.001	3.950	0.163	-0.020	0.689
Temporal	-0.208	<0.001	-0.565	0.774	0.035	0.314
**ONH parameters**						
Rim area (mm2)	0.004	0.078	0.237	0.003	-0.003	0.077
Disc area (mm2)	0.003	0.190	-0.003	0.974	<0.001	0.807
Average CDR (mm2)	-0.001	0.279	0.065	0.065	<0.001	0.227
Cup volume (mm3)	-0.001	0.085	0.003	0.003	0.001	0.164
Axial length (mm)	-0.011	0.006	0.002	0.002	0.001	0.648
**16 sections (μm)**						
TU1	-0.164	0.003	-0.539	0.784	0.032	0.360
TU2	-0.198	0.014	4.199	0.134	0.006	0.902
ST2	-0.193	0.069	10.527	0.005	-0.084	0.205
ST1	-0.388	<0.001	1.272	0.737	-0.018	0.784
SN1	-0.232	0.048	6.010	0.144	-0.080	0.273
SN2	-0.175	0.081	5.853	0.094	-0.081	0.191
NU2	-0.124	0.164	1.581	0.612	-0.025	0.648
NU1	-0.035	0.602	3.296	0.155	-0.045	0.274
NL1	0.077	0.207	6.742	0.002	-0.090	0.018
NL2	-0.014	0.863	7.576	0.008	-0.091	0.076
IN2	-0.127	0.214	3.661	0.306	-0.020	0756
IN1	-0.221	0.073	2.676	0.536	0.001	0.988
IT1	-0.382	<0.001	3.988	0.286	-0.028	0.675
IT2	-0.431	<0.001	3.991	0.311	-0.010	0.885
TL2	-0.288	<0.001	-1.869	0.488	0.044	0.355
TL1	-0.164	<0.001	-3.129	0.050	0.046	0.102

Supplemental table 2. Comparisons of sectionalized RNFL thickness between male and female in 16 sections.

Segment	Female (N=3819)	Male (N=3205)	t	*P*-value
**16 sections (μm)**				
TU1	73.99±12.92	72.69±12.7	4.279	< 0.001
TU2	101.93±18.62	97.24±17.50	10.832	< 0.001
ST2	140.08±23.73	133.93±23.85	10.746	< 0.001
ST1	156.10±24.37	155.57±24.37	0.890	0.372
SN1	139.34±26.59	137.58±26.39	2.761	0.006
SN2	133.87±23.82	132.21±22.80	2.776	0.006
NU2	102.53±21.04	102.20±19.61	0.670	0.501
NU1	74.26±15.34	73.34±14.70	2.564	0.010
NL1	69.79±13.82	67.95±13.73	5.544	< 0.001
NL2	92.71±19.58	90.02±18.79	5.850	< 0.001
IN2	129.98±23.40	127.16±23.22	4.874	< 0.001
IN1	153.88±27.41	150.89±26.93	4.569	< 0.001
IT1	169.39±24.05	166.53±23.34	5.036	< 0.001
IT2	141.13±26.01	137.34±24.99	6.192	< 0.001
TL2	87.94±18.38	87.20±17.13	1.725	0.085
TL1	63.99±10.23	64.51±10.02	-2.145	0.032

Statistics presented: mean±SD; n (%)

Supplemental table 3. Comparisons of sectionalized RNFL thickness between different age groups in 16 sections.

Segment	Age					
	**<39 (N=352)**	**40-49 (N=1728)**	**50-59 (N=2095)**	**60-69 (N=1647)**	**≥70 （N=550）**	***P*-value**
**16 sections (μm)**						
TU1	74.67±12.05	74.78±12.47	73.69±12.76	72.45±12.28	69.77±14.77	< 0.001
TU2	101.68±18.17	102.31±18.09	100.24±17.83	98.02±17.89	94.28±20.32	< 0.001
ST2	139.45±23.36	141.50±23.57	137.85±22.82	134.29±24.11	128.69±26.39	< 0.001
ST1	158.05±23.60	160.89±23.52	156.92±23.81	151.39±24.08	146.64±26.30	< 0.001
SN1	140.65±27.98	142.51±25.93	138.92±25.97	135.21±26.35	130.78±25.98	< 0.001
SN2	134.88±25.08	136.46±22.73	133.47±23.05	130.58±23.52	126.50±22.63	< 0.001
NU2	104.53±20.70	103.57±19.56	102.75±19.98	101.46±21.41	98.37±21.25	< 0.001
NU1	74.70±14.52	74.63±14.27	74.24±15.01	73.12±15.73	71.12±15.94	< 0.001
NL1	68.85±13.28	69.45±12.97	69.15±13.54	68.54±14.32	67.33±16.14	0.022
NL2	91.84±18.74	93.08±18.71	91.66±18.68	90.16±19.69	87.85±21.49	< 0.001
IN2	129.32±22.10	130.10±22.66	128.69±22.98	127.72±23.92	124.21±25.23	< 0.001
IN1	153.52±25.45	153.95±27.33	152.64±26.79	151.36±27.62	146.24±28.94	< 0.001
IT1	171.16±23.51	172.12±22.89	168.57±22.88	164.70±23.59	157.46±27.31	< 0.001
IT2	144.21±25.77	144.44±25.36	140.71±24.71	134.60±25.05	128.85±27.83	< 0.001
TL2	90.42±18.29	89.99±17.32	88.52±17.87	85.30±17.23	82.10±20.25	< 0.001
TL1	65.44±9.80	65.11±9.64	64.59±10.21	63.59±9.81	61.44±12.45	< 0.001

Statistics presented: mean±SD; n (%)

Supplemental table 4. Comparisons of sectionalized RNFL thickness between male and female.

Statistics presented: mean±SD; n (%)

Segment	Female (N=3819)	Male (N=3205)	t	*P*-value
**RNFL thickness (µm)**				
Average	114.43±10.96	112.32±10.72	8.120	**< 0.001**
Superior	142.30±18.69	139.96±18.24	5.294	**< 0.001**
Nasal	84.87±15.59	83.39±14.90	4.046	**< 0.001**
Inferior	148.63±17.74	145.50±17.37	7.447	**< 0.001**
Temporal	81.94±12.77	80.43±12.23	5.057	**< 0.001**
**ONH parameters**				
Rim area (mm2)	1.73±0.49	1.63±0.46	8.621	**< 0.001**
Disc area (mm2)	2.41±0.51	2.44±0.48	-2.244	**0.025**
Average CDR (mm2)	0.33±0.13	0.34±0.14	-5.092	**< 0.001**
Cup volume (mm3)	0.12±0.15	0.17±0.20	-11.367	**< 0.001**
Axial length (mm)	22.67±0.92	23.05±0.88	-17.805	**< 0.001**

Supplemental table 5. The supplementary analysis of VIF for the multivariate model

Variables	VIF
**Age**	1.584
**Gender**	1.815
**Smoking status**	1.768
**Body mass index**	1.050
**Diabetes**	1.048
**Coronary heart disease**	1.055
**Cataract extraction**	1.019
**Intraocular pressure**	1.031
**Spherical equivalent**	1.213
**Axial length**	1.117
**BCVA**	1.603

Supplemental table 6. Different types of OCT produced by different companies.

OCT	Time to market	Type	Company	Country	Number of standard database eyes	Race	Proportion of Asians	Is it FDA certified
Stratus OCT	2002	TD-OCT	Carl Zeiss	Germany	284	Caucasian, Asian, African, Hispanic, Indian	24%	Yes
Cirrus OCT	2007	SD-OCT	Carl Zeiss	Germany	284	Caucasian, Asian, African, Hispanic, Indian	24%	Yes
Spectralis OCT	2006	SD-OCT	Heidelberg	Germany	201	Caucasian	0%	Yes
Topcon 3D OCT	2008	SD-OCT	Topcon	Japan	800	Japanese	100%	No
RTVue OCT	2006	SD-OCT	Optovue	America	861	Caucasian, Spanish, African, Chinese, Japanese, Indian	47%	Yes

Supplemental table 7. Post hoc test for comparisons of RNFL thickness parameters among the different age groups.

	Average RNFL thickness		Superior RNFL thickness		Nasal RNFL thickness		Inferior RNFL thickness		temporal RNFL thickness	
**Comparisons**	***t***	**SE**	***t***	***P-value***	***t***	***P-value***	***t***	***P-value***	***t***	***P-value***
≥ 70 - <39 = 0	-7.753	0.728	-8.107	<0.001	3.698	0.002	-8.590	<0.001	-7.145	<0.001
40-49 - <39 = 0	0.675	0.624	1.886	0.310	0.062	1.000	0.516	0.985	-0.037	1.000
50-59 - <39 = 0	-1.271	0.614	-1.411	0.605	-0.624	0.969	-1.874	0.317	-1.817	0.348
60-69 - <39 = 0	-3.754	0.626	-4.991	<0.001	-1.853	0.328	-4.834	<0.001	-4.408	<0.001
40-49 - ≥70 = 0	8.205	0.522	13.555	<0.001	5.230	<0.001	12.592	<0.001	9.918	<0.001
50-59 - ≥70 = 0	6.259	0.511	9.853	<0.001	4.518	<0.001	9.985	<0.001	7.995	<0.001
60-69 - ≥70 = 0	3.775	0.525	5.285	<0.001	2.917	0.027	6.141	<0.001	4.648	<0.001
50-59 - 40-49 = 0	-1.946	0.347	-5.894	<0.001	-1.217	0.729	-4.250	<0.001	-3.154	0.013
60-69 - 40-49 = 0	-4.430	0.367	-11.713	<0.001	-3.264	0.009	-9.120	<0.001	-7.453	<0.001
60-69 - 50-59 = 0	-2.484	0.351	-6.431	<0.001	-2.212	0.165	-5.343	<0.001	-4.681	<0.001

Supplemental table 8. Post hoc test for comparisons of rim area RNFL thickness among age groups

	Rim area		Disc area		Average CDR		Cup volume		Axial length	
**Comparisons**	***t***	***P-value***	***t***	***P-value***	***t***	***P-value***	***t***	***P-value***	***t***	***P-value***
≥ 70 - <39 = 0	0.423	0.993	3.396	0.006	-0.271	0.999	0.116	1.000	-3.997	0.001
40-49 - <39 = 0	-0.321	0.998	0.580	0.977	-0.545	0.981	1.569	0.501	0.600	0.973
50-59 - <39 = 0	-0.298	0.998	1.893	0.306	0.370	0.996	1.586	0.490	-2.156	0.185
60-69 - <39 = 0	-0.799	0.927	2.254	0.150	0.170	1.000	1.095	0.799	-3.615	0.003
40-49 - ≥70 = 0	-0.973	0.860	-4.041	< 0.001	-0.273	0.999	1.713	0.410	6.281	<0.001
50-59 - ≥70 = 0	-0.961	0.865	-2.562	0.072	0.830	0.916	1.742	0.392	3.106	0.015
60-69 - ≥70 = 0	-1.539	0.521	-2.019	0.243	0.578	0.977	1.145	0.771	1.229	0.722
50-59 - 40-49 = 0	0.048	1.000	2.312	0.132	1.635	0.458	-0.013	1.000	-4.917	<0.001
60-69 - 40-49 = 0	-0.818	0.921	2.858	0.032	1.215	0.730	-0.798	0.927	-7.175	<0.001
60-69 - 50-59 = 0	-0.903	0.890	0.708	0.952	-0.343	0.997	-0.822	0.919	-2.681	0.053
