# Supplementary material for: Retinal nerve fibre layer thickness measured with SD-OCT in a population-based study: the Handan Eye Study
Source: Br J Ophthalmol. 2022 Apr 5;107(8):1156–64. doi: 10.1136/bjophthalmol-2021-320618 (PMC10359552; doi:10.1136/bjophthalmol-2021-320618)
Supplement: Supplementary data [file bjophthalmol-2021-320618supp002.pdf]

Point-by-point responses to the reviewers' comments:

We would like to express our sincerest appreciation to the Editor and reviewers for their efforts on our article.

Reviewer(s)' Comments to Author (if any):

Reviewer: 1

Comments to the Author

Wu et al. investigated normative profile of RNFL thickness measured by SD-OCT in Chinese population. Compared to previous studies, the number of subjects in this study is large. Also, considering the lack of studies in Asians on this topic, the current analysis is worthy of investigation. However, conclusion made in the Abstract of the paper (i.e., RNFL in the Chinese population was thicker than that in other studies) cannot be substantiated by the data. In addition, some corrections are needed to consider publication.

Major comments:

1. Presentation of the results for multivariate linear regression should be consistent throughout the manuscript.

- Abstract: result according to sex was repeated.
- Abstract and page 10: "decreased BMI" and "(BMI>30)" – These two are contradictory.
- In the abstract, thinner RNFL was significantly associated with lower SE and longer AL. However, in the Results section (page 10, line 20), thinner RNFL was significantly associated with decreased AL.

Response: We would like to thank the reviewer for the positive comments and thorough evaluation of our manuscript, we apologized for the repetitive and unclear expression leading to misunderstanding. We have modified the corresponding part. (Line 36-37, Line 210-212)

2. Some expressions need to be clarified.

- Abstract conclusion, "RNFL thickness in Chinese population is thicker compared to other studies": Since this study did not conduct a qualitative systematic review on this topic, it would be better to present conclusions on results directly obtained from the analysis.

Response: We thank the reviewer for pointing out the flaw in this expression. We apologized for the improper statement due to our negligence. We have modified in the corresponding part. (Line 40-41)

- Abstract conclusion, "Meanwhile, many ocular and systemic factors are closely related to the changes of RNFL": This study did not evaluate "changes" of RNFL.

Response: Thanks for your suggestion, we apologized for the inappropriate description. We described the true value but not the changes, which were mostly adopted in the cohort study. Thanks for your professionalism and scrutiny. We also amended the original text accordingly. (Line 42)

3. Considerations for statistics

- In the multivariate analyses, multicollinearity needs to be considered. This is because the factors included in the analysis (i.e., Diabetes and HbA1c or SE and AL) are likely to have a significant correlation with each other.

Response: Thanks for your suggestion. We supplemented the Variance Inflation Factor (VIF) for each covariate in the multivariate model. The VIFs were all less than 10, indicating no obvious multicollinearity in the reported model. The VIFs are listed as follows (as there was no HbA1c parameter included in our multivariate model analysis, thus HbA1c is not shown in the following table). We also added the relevant description in the manuscript. (Line 154, Line 213, Supplemental table 5)

The analysis of VIF for the multivariate model

Variables	VIF
**Age**	1.584
**Gender**	1.815
**Smoking status**	1.768
**Body mass index**	1.050
**Diabetes**	1.048
**Coronary heart disease**	1.055
**Cataract extraction**	1.019
**Intraocular pressure**	1.031
**Spherical equivalent**	1.213
**Axial length**	1.117
**BCVA**	1.603

- Table 2 and Supplemental Tables: OCT sectors should be compared considering issue of multiple comparisons.

Response: Thanks for your suggestion. We applied Bonferroni correction to control for the potential errors found in multiple comparisons.

The adjusted significant level was $\alpha' = \alpha/n$, in which $\alpha = 0.05$, n = 10. Then the comparisons of OCT sectors in Table 2 were considered to be statistically significant only when the P-value was less than or equal to $\alpha' = 0.005$. After the adjustment of the significant level, our conclusion regarding comparisons among age groups in Table 2 remained unchanged. Besides, we did not consider Bonferroni correction for comparisons in

supplementary tables because these variables were not the primary effects. And results from comparisons in supplemental tables were considered as explanatory rather than confirmatory conclusions.

- Table 2: post hoc test should be considered after ANOVA to reveal difference between each age group.

Response: Thanks for your suggestion. We supplemented Turkey test as a post hoc test after ANOVA across age groups. The whole results are listed as follows, and we have added the integrated version as supplemental tables 7 and 8. Also, the accordingly description in the method and result part was added in the manuscript. (Line 152, 188)

Table. Post hoc test for comparisons of average RNFL thickness among age groups

Comparisons	Estimate	SE	t	P-value
**≥ 70 - <39 = 0**	-7.753	0.728	-10.346	<0.001
**40-49 - <39 = 0**	0.675	0.624	1.083	0.805
**50-59 - <39 = 0**	-1.271	0.614	-2.069	0.221
**60-69 - <39 = 0**	-3.754	0.626	-5.996	<0.001
**40-49 - ≥70 = 0**	8.205	0.522	15.718	<0.001
**50-59 - ≥70 = 0**	6.259	0.511	12.252	<0.001
**60-69 - ≥70 = 0**	3.775	0.525	7.190	<0.001
**50-59 - 40-49 = 0**	-1.946	0.347	-5.616	<0.001
**60-69 - 40-49 = 0**	-4.430	0.367	-12.064	<0.001
**60-69 - 50-59 = 0**	-2.484	0.351	-7.073	<0.001

Table. Post hoc test for comparisons of superior RNFL thickness among age groups

Comparisons	Estimate	SE	t	P-value
**≥70 - <39 = 0**	-10.061	1.241	-8.107	<0.001
**40-49 - <39 = 0**	2.005	1.063	1.886	0.310
**50-59 - <39 = 0**	-1.478	1.047	-1.411	0.605
**60-69 - <39 = 0**	-5.329	1.068	-4.991	<0.001
**40-49 - ≥70 = 0**	12.066	0.890	13.555	<0.001
**50-59 - ≥70 = 0**	8.583	0.871	9.853	<0.001
**60-69 - ≥70 = 0**	4.732	0.895	5.285	<0.001
**50-59 - 40-49 = 0**	-3.483	0.591	-5.894	<0.001
**60-69 - 40-49 = 0**	-7.334	0.626	-11.713	<0.001
**60-69 - 50-59 = 0**	-3.851	0.599	-6.431	<0.001

Table. Post hoc test for comparisons of nasal RNFL thickness among age groups

Comparisons	Estimate	SE	t	P-value
**≥70 - <39 = 0**	-3.857	-1.043	3.698	0.002
**40-49 - <39 = 0**	0.055	0.893	0.062	1.000
**50-59 - <39 = 0**	-0.549	0.880	-0.624	0.969

**60-69 - <39 = 0**	-1.662	0.897	-1.853	0.328
**40-49 - ≥70 = 0**	3.912	0.748	5.230	<0.001
**50-59 - ≥70 = 0**	3.307	0.732	4.518	<0.001
**60-69 - ≥70 = 0**	2.194	0.752	2.917	0.027
**50-59 - 40-49 = 0**	-0.604	0.496	-1.217	0.729
**60-69 - 40-49 = 0**	-1.717	0.526	-3.264	0.009
**60-69 - 50-59 = 0**	-1.113	0.503	-2.212	0.165

Table. Post hoc test for comparisons of inferior RNFL thickness among age groups

Comparisons	Estimate	SE	t	P-value
**≥70 - <39 = 0**	-10.213	1.189	-8.590	<0.001
**40-49 - <39 = 0**	0.526	1.019	0.516	0.985
**50-59 - <39 = 0**	-1.880	1.003	-1.874	0.317
**60-69 - <39 = 0**	-4.945	1.023	-4.834	<0.001
**40-49 - ≥70 = 0**	10.738	0.853	12.592	<0.001
**50-59 - ≥70 = 0**	8.333	0.835	9.985	<0.001
**60-69 - ≥70 = 0**	5.268	0.858	6.141	<0.001
**50-59 - 40-49 = 0**	-2.406	0.566	-4.250	<0.001
**60-69 - 40-49 = 0**	-5.470	0.600	-9.120	<0.001
**60-69 - 50-59 = 0**	-3.065	0.574	-5.343	<0.001

Table. Post hoc test for comparisons of temporal RNFL thickness among age groups

Comparisons	Estimate	SE	t	P-value
**≥70 - <39 = 0**	-6.076	0.850	-7.145	<0.001
**40-49 - <39 = 0**	-0.027	0.729	-0.037	1.000
**50-59 - <39 = 0**	-1.304	0.718	-1.817	0.348
**60-69 - <39 = 0**	-3.224	0.732	-4.408	<0.001
**40-49 - ≥70 = 0**	6.049	0.610	9.918	<0.001
**50-59 - ≥70 = 0**	4.772	0.597	7.995	<0.001
**60-69 - ≥70 = 0**	2.852	0.614	4.648	<0.001
**50-59 - 40-49 = 0**	-1.277	0.405	-3.154	0.013
**60-69 - 40-49 = 0**	-3.197	0.429	-7.453	<0.001
**60-69 - 50-59 = 0**	-1.921	0.410	-4.681	<0.001

Table. Post hoc test for comparisons of rim area RNFL thickness among age groups

Comparisons	Estimate	SE	t	P-value
**≥70 - <39 = 0**	0.014	0.033	0.423	0.993
**40-49 - <39 = 0**	-0.009	0.028	-0.321	0.998
**50-59 - <39 = 0**	-0.008	0.028	-0.298	0.998
**60-69 - <39 = 0**	-0.022	0.028	-0.799	0.927
**40-49 - ≥70 = 0**	-0.023	0.023	-0.973	0.860
**50-59 - ≥70 = 0**	-0.022	0.023	-0.961	0.865

**60-69 - ≥70 = 0**	-0.036	0.024	-1.539	0.521
**50-59 - 40-49 = 0**	0.001	0.016	0.048	1.000
**60-69 - 40-49 = 0**	-0.013	0.016	-0.818	0.921
**60-69 - 50-59 = 0**	-0.014	0.016	-0.903	0.890

Table. Post hoc test for comparisons of disc area RNFL thickness among age groups

Comparisons	Estimate	SE	t	P-value
**≥70 - <39 = 0**	0.114	0.034	3.396	0.006
**40-49 - <39 = 0**	0.017	0.029	0.580	0.977
**50-59 - <39 = 0**	0.054	0.028	1.893	0.306
**60-69 - <39 = 0**	0.065	0.029	2.254	0.150
**40-49 - ≥70 = 0**	-0.098	0.024	-4.041	< 0.001
**50-59 - ≥70 = 0**	-0.061	0.024	-2.562	0.072
**60-69 - ≥70 = 0**	-0.049	0.024	-2.019	0.243
**50-59 - 40-49 = 0**	0.037	0.016	2.312	0.132
**60-69 - 40-49 = 0**	0.049	0.017	2.858	0.032
**60-69 - 50-59 = 0**	0.012	0.016	0.708	0.952

Table. Post hoc test for comparisons of average CDR RNFL thickness among age groups

Comparisons	Estimate	SE	t	P-value
**≥70 - <39 = 0**	-0.002	0.009	-0.271	0.999
**40-49 - <39 = 0**	-0.004	0.008	-0.545	0.981
**50-59 - <39 = 0**	0.003	0.008	0.370	0.996
**60-69 - <39 = 0**	0.001	0.008	0.170	1.000
**40-49 - ≥70 = 0**	-0.002	0.007	-0.273	0.999
**50-59 - ≥70 = 0**	0.005	0.006	0.830	0.916
**60-69 - ≥70 = 0**	0.004	0.007	0.578	0.977
**50-59 - 40-49 = 0**	0.007	0.004	1.635	0.458
**60-69 - 40-49 = 0**	0.006	0.005	1.215	0.730
**60-69 - 50-59 = 0**	-0.002	0.004	-0.343	0.997

Table. Post hoc test for comparisons of cup volume RNFL thickness among age groups

Comparisons	Estimate	SE	t	P-value
**≥70 - <39 = 0**	0.001	0.012	0.116	1.000
**40-49 - <39 = 0**	0.016	0.010	1.569	0.501
**50-59 - <39 = 0**	0.016	0.010	1.586	0.490
**60-69 - <39 = 0**	0.011	0.010	1.095	0.799
**40-49 - ≥70 = 0**	0.015	0.009	1.713	0.410
**50-59 - ≥70 = 0**	0.015	0.008	1.742	0.392
**60-69 - ≥70 = 0**	0.010	0.009	1.145	0.771
**50-59 - 40-49 = 0**	<0.001	0.006	-0.013	1.000
**60-69 - 40-49 = 0**	-0.005	0.006	-0.798	0.927

**60-69 - 50-59 = 0**	-0.005	0.006	-0.822	0.919

Table. Post hoc test for comparisons of axial length RNFL thickness among age groups

Comparisons	Estimate	SE	t	P-value
**≥70 - <39 = 0**	-0.256	0.064	-3.997	0.001
**40-49 - <39 = 0**	0.033	0.055	0.600	0.973
**50-59 - <39 = 0**	-0.116	0.054	-2.156	0.185
**60-69 - <39 = 0**	-0.199	0.055	-3.615	0.003
**40-49 - ≥70 = 0**	0.288	0.046	6.281	<0.001
**50-59 - ≥70 = 0**	0.140	0.045	3.106	0.015
**60-69 - ≥70 = 0**	0.057	0.046	1.229	0.722
**50-59 - 40-49 = 0**	-0.149	0.030	-4.917	<0.001
**60-69 - 40-49 = 0**	-0.232	0.032	-7.175	<0.001
**60-69 - 50-59 = 0**	-0.083	0.031	-2.681	0.053

4. Discussion Page 11 line 19-55: The authors claimed that the type of SD-OCT device and age distribution might be the main reasons for RNFL thickness difference. However, in this study, it is presumed that not only these two factors but also axial length had a significant effect on RNFL thickness measurement. It should be further discussed in the manuscript.

Response: Thanks for your suggestion. The age and type of SD-OCT were not only the primary factors affecting RNFL. There were a lot of factors affecting RNFL thickness, which were further discussed and analyzed in this study, we revised and clearly stated in the text (the axial length was discussed on Line 317-324).

5. The average AL was about 22.8 mm, which is much shorter compared with previous study on Chinese population. This needs to be explained.

Ex) Ho et al. Ophthalmology 2019 (reference #53)

- Chinese (n=1371 participants)
- Average RNFL thickness: 95.7
- Average age: 54.7
- Average axial length: 24.0

Response: Thanks for your advice on this study. Most of the subjects in the study by Ho et al. were urban population, while the participants in this study were rural population. Relatively speaking, the myopia proportion induced by the education level and social factors of the rural population were lower than that of the urban population, so the axial length was relatively short, while axial length was also an important influence factor for the evaluation of RNFL thickness, so the relatively short axial length in this study can result in a thicker RNFL as a whole. This factor should be taken into account. Thank you for finding the shortcomings of this study, and we have added the explanation of this part in an appropriate section of Limitation (Lines 352-357).

6. Page 10 line 40: "We demonstrated that…. Using SD-OCT": this sentence cannot be substantiated by the data presented in the Methods/Results sections. I believe that this conclusion cannot be draw from a simple comparison with the numbers presented in previous studies.

Response: Thanks for your careful observation and suggestion on the rigor of the manuscript. This study did not make a statistical comparison between Asian and European populations in RNFL thickness, so the conclusion drawn was inaccurate. We just got the information from the observation of statistical results, so we modified the expression of conclusion. It was inaccurate to use the European Standard database when applying OCT to measure Asian population (Line 222-225).

Minor comments:

1. 62 references are indicated in the Supplement file, but only 35 are indicated in the reference lists in the manuscript.

Response: Thank you for your question. Given the submission requirements of the journal, our study controlled references of the whole article within 35, so the references in other supplementary documents were listed only in the supplementary references. We put all the references together and relist. If the editor has any special requirements, we will further modify as required by the editor (see References).

2. Abbreviations and acronyms should be consistently used after their first appearance. Ex) AL for axial length.

Response: Thank you for your suggestion on the shortcomings of this study. We carefully checked the use of abbreviations and acronyms in the whole paper and made a targeted modification in the manuscript.

3. Page 8 line 31: The authors mentioned that the RNFL thickness satisfies the "ISNT rule", which is generally used to describe the relative thickness of the neuroretinal rim.

Response: We thank the reviewer for pointing out this issue. This is where we neglect and state improperly. We have amended the original text accordingly.

4. Page 15 line 36: It would be better to separate the limitation part as an independent paragraph.

Response: Thanks for your suggestion. The limitation was an important part of the discussion. We highly appreciate your suggestion and set out Limitation as an individual paragraph (Line 352).

Reviewer: 2

Comments to the Author

This study investigated the normative profile of retinal nerve fiber layer (RNFL) thickness based on spectral-domain optical coherence tomography and its associations with related parameters in a Chinese population.

Overall, this study supports the clinically important finding that the Chinese have thicker peripapillary RNFL from a population study, although this is not a novel one.

Specific comments:

1. 4th sentence, Abstract needs to be rephrased:
   1) delete either male or male sex,
   2) change 'less diabetes' to 'absence of diabetes,
   3) change 'more history of cataract surgery' to ' history of cataract surgery'.

Response: Thanks for the meticulous concern, we apologized for the repetitive and unclear expression leading to misunderstanding. The relevant mistakes in spelling and punctuation have been addressed. (Line 36-38, Line 210-212)

2. Proof-reading and editing by a professional native English speaker is needed. Grammar errors are noted throughout the manuscript: e.g. Line 15-28, Introduction: With the developing of SD-OCT gradually, automated segmentation and measurement of individual retinal layers is ...

Response: We thank the reviewer for the comment, we have amended the relevant sentence in the manuscript. (Line 75-78) And accordingly, we already went through the manuscript and corrected and polished the language mistakes in the revised manuscript. The revised manuscript has been edited and proofread by a native speaker.

3. A separate paragraph needs to be added for better discussion of the association between cataract surgery and RNFL thickness.

Response: Thank you for your suggestions on this study. According to the suggestion, we queried the relationship between cataract surgery and RNFL thickness and found some valuable opinions also hypotheses. The contents were listed as an individual paragraph and discussed accordingly. (Lines 333-343).

4. Some OCT machines are known to include Asian eyes in their normative database. Please provide a table or a paragraph comparing the percentages of Asians in the normative database in different recent OCT machines.

Response: Thank you for your suggestion on this study. According to your suggestion, we

summarized the common OCTs that were commercially available, and searched for information in RNFL thickness standard database related to instruments from the official website and related literature of OCT and compiled them into a table (Supplementary Table 6). As can be seen from these results, the RNFL thickness standard database of OCT instruments produced by different companies varied greatly from population to population. Hence, it is necessary to list and provide clinical reference for the measurement of RNFL thickness and the diagnosis of relevant diseases (Line 251-261).

Editor(s)' Comments to Author (if any):

Section Editor
Comments to the Author:
(There are no comments.)

We thank the reviewer for pointing out this issue. This is really where we neglect and state improperly.
